# Audit of the ECT Service in Department of Psychiatry and Behavioural Scinences DHQ Hospital Faisalabad Against the National Institute of Clinical Excellence (NICE) Guidelines

**DOI:** 10.1192/bjo.2025.10578

**Published:** 2025-06-20

**Authors:** Duaa Fatima, Imtiaz Ahmad Dogar, Palvisha Sajid, Sammar Fatima

**Affiliations:** Faisalabad Medical University, Faisalabad, Pakistan

## Abstract

**Aims:** The aim of the audit was to ensure that the referral practices and assessment methods of patients who received ECT were carried out and documented as per the NICE guidelines.

**Methods:** Study was carried out at Allied Hospital 2, Faisalabad Medical University, Faisalabad. Data was collected from May 2023 to October 2023. Study includes all the inpatients referred for ECT during a 6-month period from May 2023 to October 2023. The data was collected by examination of the patient’s notes to establish the adherence to all 9 standards required by NICE guidance.

**Results:** Total number of patients was 47 (24 females and 23 males) the results show that ECT guidelines were followed and 100% compliance was achieved in all standards except one. Assessment of cognitive functions, 44 patients (86.3%) were assessed for their cognitive functions before the course of treatment but only 29 patients (56.9%) were assessed after the course.

**Conclusion:** The adherence to referral and consent standards was excellent; however, greater attention is needed in the implementation and documentation of cognitive assessments before, during, and after treatment. This is particularly important due to the high incidence of cognitive dysfunction observed following ECT administration.

